# *Alistipes* Bacteremia in Older Patients with Digestive and Cancer Comorbidities, Japan, 2016–2023

**DOI:** 10.3201/eid3104.241284

**Published:** 2025-04

**Authors:** Naoki Watanabe, Tomohisa Watari, Naoto Hosokawa, Yoshihito Otsuka

**Affiliations:** Kameda Medical Center, Chiba, Japan

**Keywords:** bacteremia, bacteria, Alistipes, anaerobic bacteria, microbial sensitivity tests, antimicrobial resistance, blood culture, infections, Japan

## Abstract

The clinical characteristics of *Alistipes* bacteremia remain insufficiently understood. We retrospectively analyzed 13 cases of *Alistipes* bacteremia at a tertiary care center in Japan. Of the 13 patients, 7 were male and 6 female; 10 were >65 years of age. Of 9 patients with comorbidities, 7 had solid tumors or hematological malignancies and 11 had gastrointestinal symptoms. Isolates identified were *Alistipes finegoldii* in 4 cases, *A. onderdonkii* in 4, *A. putredinis* in 3, *A. indistinctus* in 2, and *A. ihumii* in 1. Ten strains exhibited low MICs against β-lactam/β-lactamase inhibitors and metronidazole. We observed high MICs against penicillin, ceftriaxone, and minocycline. Several strains harbored antimicrobial resistance genes, including *adeF*, *tet(Q)*, *cfxA3*, *cfxA4*, and *ermG*. Twelve patients received β-lactam/β-lactamase inhibitors; 2 patients with solid tumors experienced septic shock and died. *Alistipes* bacteria can translocate from the gastrointestinal tract into the bloodstream, particularly in cases of inflammation, obstruction, or perforation, leading to severe infections.

*Alistipes* is an anaerobic bacterium rarely detected in clinical samples. Initially described as a bile-resistant, pigment-producing, gram-negative bacillus (*1*,*2*), *Alistipes* was classified as a new genus in 2003 (*3*). The genus contains 11 species, including *A. finegoldii* (*3*), *A. putredinis* (*3*), and *A. onderdonkii* (*4*), which have been isolated from the appendix, feces, intra-abdominal fluid, and blood (*3*,*5*–*7*). Previous studies have indicated that the bacteria may contribute both protectively and pathogenically to various diseases (*8*). Anaerobic bacteria, which are part of the normal human flora, are also significant pathogens in bacteremia and other infectious diseases. Anaerobic bacteremia is often associated with abdominal, pelvic, skin, and soft tissue infections (*9*). *Alistipes* is associated with infections such as appendicitis (*1*,*2*) and intra-abdominal infections (*10*). Three cases of *Alistipes* bacteremia in patients experiencing colon cancer (*11*) or peritonitis (*12*) have been reported. Because reports of *Alistipes* bacteremia are rare, the clinical characteristics of *Alistipes* bacteremia remain insufficiently described.

Inadequate treatment of anaerobic bacterial infections can lead to more serious illness. In cases of anaerobic bacteremia, the mortality rate is higher in patients receiving ineffective antimicrobial therapy than in those receiving appropriate antimicrobial therapy (*13*). Therefore, understanding the antimicrobial susceptibility of *Alistipes* species is crucial for implementing effective treatments. Of the 3 *Alistipes* strains isolated from the blood, 2 have shown low susceptibility to penicillin and 1 to cefotetan (*11*,*12*). As of January 2025, the antimicrobial susceptibility of *Alistipes* isolates from the blood is not well understood. Therefore, this study aimed to investigate and describe the clinical characteristics of *Alistipes* bacteremia in a tertiary care center in Japan. In addition, we evaluated the accuracy of identification testing for *Alistipes* species and assessed the antimicrobial resistance of the isolates. The Ethics Committee of Kameda Medical Center approved this study (agreement no. 23–121). The requirement for written informed consent was waived because patient data were anonymized.

## Materials and Methods

### Study Design and Data Collection

We conducted a retrospective cohort study at Kameda Medical Center in Japan during April 2016–December 2023. We reviewed 31,875 patients who underwent blood cultures and identified *Alistipes* cases. In cases where multiple positive blood cultures were obtained, we considered them part of the same culture-positive episode unless clinical evidence indicated the presence of a novel infection. We included cases in which a new infection was clinically identified as discrete culture-positive episodes within the investigation. We collected patient data and microbiologic testing results from medical records and a laboratory system. Patient data included age, sex (male or female), infection type (community-acquired, healthcare-associated), comorbidities, symptoms, infection sites, procedures, antimicrobial therapy, hospitalization days from blood collection, and outcomes. We defined healthcare-associated infections as cases with 1 of the following conditions: onset after 48 hours of hospitalization or patients receiving medical care, such as a nursing home, dialysis, or outpatient chemotherapy.

### Blood Culture and Identification Testing

Blood cultures were performed using BD Bactec FX Blood Culture System (BD, https://www.bd.com). Blood samples were collected using Plus Aerobic and Lytic Anaerobic Media for adult patients and Peds Plus Medium for pediatric patients (all from BD). The blood cultures were incubated at 35°C for 7 days. The number of positive blood culture sets, time to positivity, and detected *Alistipes* were recorded. *Alistipes* strains were stored at –80°C for identification, whole-genome sequencing (WGS), and antimicrobial susceptibility testing. The preserved strains were cultured anaerobically using Brucella HK agar medium (Kyokuto Pharmaceutical Industrial, https://www.kyokutoseiyaku.co.jp) at 35°C for 2 days and were used for each test.

The strains were identified using matrix-assisted laser desorption/ionization time-of-flight (MALDI-TOF) mass spectrometry. MALDI-TOF mass spectrometry was performed using the MALDI Biotyper system, which included the microflex LT/SH and flexControl version 3.4 (Bruker Daltonics GmbH, https://www.bruker.com). *Alistipes* colonies that developed on the agar medium were selected and applied to the test plate. Mass spectra were analyzed using the MALDI Biotyper Library, which recorded the highest score for the predicted species. Identification results were accepted at the species level for scores of >2.0. In cases for which the standard cell smear method could not identify the species, the strains were retested using the formic acid on-plate or formic acid extraction method.

### Polymerase Chain Reaction and Whole-Genome Analysis

We validated the identification results by 16S rRNA gene and whole-genome analyses of the preserved strains; we excluded nonpreserved strains from our analysis. We extracted genomic DNA from the strains using magLEAD and consumable kits (Precision System Science, https://www.pss.co.jp). We used GeneAtlas Type G (Astec, https://www.astec-corp.co.jp) as the thermal cycler and Premix Taq Hot Start Version (Takara Bio, https://www.takara-bio.com) for the PCR reagent. We performed PCR in accordance with previously reported primers for the 16S rRNA gene (*14*–*16*) (Appendix Table 1). We used FASMAC (https://fasmac.co.jp), for sequencing and EzBioCloud (*17*) to analyze the resulting sequences for species identification. We used Geneious Prime 2024.0.5 (Geneious, https://www.geneious.com) to analyze the 16S rRNA gene sequences and construct a phylogenetic tree. We performed 16S rRNA gene reanalysis on 3 *A. finegoldii* strains (302398, 4401054, and Granada) from previous bacteremia cases (*11*,*12*) and analyzed them using the same methods as on the strains we collected in this study.

We prepared fragment libraries for whole-genome analysis by using Illumina DNA Prep Tagmentation (M) Beads (Illumina, https://www.illumina.com). We performed sequencing with the MiSeq Reagent Micro Kit (Illumina) to generate paired-end reads and conducted de novo assembly by using CLC Genomics Workbench version 22.0.2 (QIAGEN, https://www.qiagen.com). We used CheckM (*18*) as a quality check for the genome and excluded samples with a contamination rate >5% from subsequent analysis. We used DFAST (*19*) to obtain average nucleotide identity (ANI), identifying bacterial species with a threshold of >95%. For subspecies analysis, we performed DNA-DNA hybridization (DDH) analysis using Genome–Genome Distance Calculator 3.0 (https://ggdc.dsmz.de) as described previously (*20*). We set the threshold for defining subspecies at 79% DDH of the previously reported criteria for subspecies classification (*21*). We analyzed the presence of antimicrobial-resistance genes using the Comprehensive Antibiotic Resistance Database (*22*).

### Antimicrobial Drug Susceptibility Testing

We evaluated antimicrobial drug susceptibility of the preserved strains using the broth microdilution method with brucella broth and dry plates (Eiken Chemical, https://www.eiken.co.jp). We prepared the inoculum according to the manufacturer’s instructions and transferred to a microplate at a final volume of ≈1 × 10^5^ CFU per well. After inoculation, we incubated the microplate anaerobically at 35°C–37°C for 46–48 hours. We determined MICs after confirming bacterial growth in the control wells. The dry plate contained the following antimicrobial drugs: penicillin (PEN), ampicillin/sulbactam (SAM), amoxicillin/clavulanic acid (AMC), piperacillin/tazobactam (TZP), ceftriaxone (CRO), cefoxitin (FOX), imipenem (IPM), clindamycin (CLI), minocycline (MINO), moxifloxacin (MXF), and metronidazole (MTZ). After determining the MICs, we calculated the concentrations required to inhibit 50% (MIC_50_) and 90% (MIC_90_) of the strains.

### Statistical Analyses

We summarized the characteristics of *Alistipes* cases using EZR version 1.54, as described (*23*). We reported continuous variables as medians and interquartile ranges (IQRs) and categorical variables as actual numbers. We calculated the proportion of patients with *Alistipes* bacteremia among all patients who underwent blood culture using 95% CIs. We generated survival curves using the Kaplan–Meier method and estimated 30-day survival rates using the log-rank test. We reported time to positivity as the median and IQR, excluding the results from blood culture bottles with multiple pathogens.

## Results

### Patient Characteristics and Clinical Courses

We confirmed that, over a 7-year, 9-month period, 13 cases of *Alistipes* bacteremia were identified, representing 0.04% (95% CI 0%–0.1%) of all patients who underwent blood cultures (Table 1). No cases of multiple culture-positive episodes or relapses were observed. Median age of the patients was 72 years (IQR 65–85 years). Of the 13 patients, 7 were male and 6 female; 10 were >65 years of age. Nine patients had comorbid conditions with a Charlson Comorbidity Index score >1. Six patients had solid tumors, and 1 patient had hematological malignancy.

**Table 1:** Demographic and clinical characteristics of patients with *Alistipes* bacteremia in a tertiary care center in Japan, 2016–2023*

Characteristics	All, n = 13	Alive, n = 11	Death, n = 2
Age			
Median, y (IQR)	72 (65–85)	70 (59–85)	80 (78–83)
>65 y	10 (76.9)	8 (72.7)	2 (100.0)
Sex			
F	7 (53.8)	6 (54.5)	1 (50.0)
M	6 (46.2)	5 (45.5)	1 (50.0)
Site of acquisition			
Community	7 (53.8)	7 (63.6)	0
Healthcare-associated	6 (46.2)	4 (36.4)	2 (100.0)
Charlson Comorbidity Index score			
0	4 (30.8)	4 (36.4)	0
1–2	4 (30.8)	3 (27.3)	1 (50.0)
3–4	2 (15.4)	2 (18.2)	0
>5	3 (23.1)	2 (18.2)	1 (50.0)
Comorbid conditions			
Solid tumor	6 (46.2)	4 (36.4)	2 (100.0)
Hematologic malignancies	1 (7.7)	1 (9.1)	0
Leukopenia	2 (15.4)	2 (18.2)	0
Chemotherapy	3 (23.1)	3 (27.3)	0
Immunosuppressive therapy	0	0 (0.0)	0
Intravascular device	3 (23.1)	2 (18.2)	1 (50.0)
Symptoms			
Abdominal pain	9 (69.2)	8 (72.7)	1 (50.0)
Diarrhea	1 (7.7)	1 (9.1)	0
Sepsis or septic shock	3 (23.1)	1 (9.1)	2 (100.0)
Fever	6 (46.2)	6 (54.5)	0
Vomiting	3 (23.1)	3 (27.3)	0
Time to positivity, h (IQR)	81 (71–106)	76 (68–103)	94 (88–99)

*Values are no. (%) except as indicated. IQR, interquartile range.

The most common clinical sign/symptom was abdominal pain (9 patients), followed by fever (6 patients) (Table 2). Of the 13 case-patients, 11 experienced gastrointestinal symptoms, including abdominal pain, vomiting, diarrhea, abdominal distention, or some combination. With the exception of 1 patient who experienced abdominal distention, all patients reported the onset of new gastrointestinal symptoms within 3 days before or after the collection of blood cultures. Median time to positivity was 81 hours (IQR 71–106 hours); *Alistipes* strains from 3 cases were detected in blood cultures that tested positive >120 hours after the start of incubation. 

**Table 2:** Patient characteristics and clinical course of *Alistipes* bacteremia in a tertiary care center, Japan, 2016–2023*

Case ID	Age, y/sex	Comorbid conditions	Clinical symptoms	Clinical diagnosis	Therapy†	Outcome (time)
1	80s/F	Hypertension, diabetes mellitus, lipid disorder, cerebral infarction history, rectal cancer	Abdominal pain, septic shock	Colon perforation, colonic obstruction	TZP and vancomycin (1 d)	Death (1 d)
**2**	50s/M	None	Abdominal pain	Acute appendicitis	SAM (3 d), AMC (11 d), appendectomy	Alive, discharged (6 d)
3	50s/F	Ovarian cancer, febrile neutropenia	Abdominal pain, fever	Perforated colon	SAM (24 d), TZP (12 d)	Alive, discharged (50 d)
4	90s/F	Alzheimer’s-type dementia, Hypertension, epilepsy, chronic constipation	Abdominal pain, vomiting, diarrhea, fever	Acute appendicitis, secondary peritonitis, paralytic ileus, aspiration pneumonia	SAM (23 d), AMC (5 d)	Alive, discharged (22 d)
**5**	60s/F	Diabetes mellitus, colon cancer	Abdominal pain, septic shock	Generalized peritonitis, intestinal perforation, Nonocclusive mesenteric ischemia, ovarian necrosis	TZP (2 d), cefmetazole (17 d), colectomy, small bowel resection, bilateral salpingo-oophorectomy	Alive, transferred (114 d)
6	70s/M	Febrile neutropenia, previous hepatitis B virus infection, hypertension, diabetes mellitus, benign prostatic hyperplasia, acute myeloid leukemia	Fever, abdominal pain	Colonic diverticulitis	Cefepime and MTZ (10 d), SAM and MTZ (3 d)	Alive, discharged (228 d)
7	30s/M	Epilepsy, under treatment for CRBSI with *Staphylococcus aureus*	Fever	Bacteremia of unknown origin	Cefazolin (5 d), SAM (12 d)	Alive, transferred (41 d)
8	70s/M	Oral candida, adenocarcinoma (primary site unknown)	Septic shock, ascites, abdominal distension‡	Bowel obstruction	TZP (1 d), TZP and fluconazole (1 d)	Death (1 d)
**9**	60s/F	Rectal cancer	Abdominal pain, vomiting	Colon perforation, peritonitis	TZP (1 d), SAM (14 d), Hartmann’s procedure	Alive, discharged (28 d)
**10**	90s/F	Hypertension, dyslipidemia, osteoporosis, postoperative Mallory–Weiss syndrome, suspected aspiration pneumonia	Fever, tachycardia	Bowel obstruction	Cefotiam (4 d), SAM (15 d), intestinal resection	Alive, discharged (44 d)
**11**	80s/F	Osteoporosis, dyslipidemia	Abdominal pain	Perforated appendicitis, peritonitis, intra-abdominal abscess	SAM (11 d), SAM and MTZ (8 d), appendectomy	Alive, discharged (19 d)
12	70s/M	Cerebral infarction history, cholecystectomy history	Chills, fever, vomiting	Bacteremia of unknown origin	Ciprofloxacin and MTZ (3 d), ceftriaxone and MTZ (10 d)	Alive (outpatient)
**13**	80s/M	Hypertension, cholecystectomy history, rectal cancer	Abdominal pain	Colorectal perforation	SAM (10 d), TZP (12 d), Hartmann procedure	Alive, discharged (53 d)

*Bold text indicates cases in which multiple pathogens were detected as follows: 2, *Alistipes onderdonkii*, *Bacteroides uniformis*, *Escherichia coli*, and *Pseudomonas nitroreducens*; 5, *A. putredinis* and *E. coli*; 9, *A. putredinis* and *Flavonifractor plautii*; 10, *A. indistinctus* and *Klebsiella pneumoniae*; 11,  *A. onderdonkii* and *B. salyersiae*; 13, *A. finegoldii* and *A. ihumii*. AMC, amoxicillin/clavulanic acid; CRBSI, catheter-related bloodstream infection; ID, identification; MTZ, metronidazole; SAM, ampicillin/sulbactam; transferred, transferred to another hospital; TZP, piperacillin/tazobactam. †Numbers in parentheses indicate duration of drug treatment. Most patients received multiple antimicrobial drugs; shown are the 2 used for the  longest duration. ‡Patient had been experiencing abdominal distention caused by accumulation of ascites even before the onset of bacteremia.

Eleven of 13 patients had gastrointestinal tract disease, and the site of infection was in the gastrointestinal tract. The remaining 2 patients (cases 7 and 12) had *Alistipes* bacteremia with unknown infection sites. The patient in case 7 experienced a fever during treatment for a catheter-related bloodstream infection caused by *Staphylococcus aureus*; *A. indistinctus* was detected in a blood culture collected at the time of fever. The patient in case 12 had a history of cerebral infarction and cholecystectomy; that patient sought care for chills, fever, and vomiting, and blood cultures were positive for *A. putredinis*.

All patients received clinically diagnoses of true bacteremia and received antimicrobial therapy (duration 13–36 days). The most prescribed antimicrobial drugs were β-lactam/β-lactamase inhibitors (BLBLIs); 9 patients received SAM, and 6 patients received TZP. Six patients exhibited polymicrobial bacteremia; the predominant co-pathogens were human intestinal bacteria. The co-pathogens were 2 cases of *Escherichia coli*, 1 of *Klebsiella pneumoniae*, 2 of *Bacteroides* spp., 2 of other anaerobic bacteria (n = 2), and 1 of *Pseudomonas* species.

### Clinical Outcomes

Of the 13 patients, 11 achieved clinical resolution after antimicrobial therapy, whereas the remaining 2 (cases 1 and 8) died. The estimated 30-day survival rate for *Alistipes* bacteremia was 84.6% (95% CI 51.2%–95.9%). 

The patient in fatal case 1 was a woman in her 80s with rectal cancer and liver metastases who was admitted to the hospital for abdominal pain. Upon arrival at the hospital, she experienced septic shock; she received intravenous fluids, blood cultures were obtained, and TZP and VCM were administered. Her condition briefly stabilized after the drug infusion but deteriorated with the development of acidosis, hyperkalemia, and elevated lactic acid levels. The patient received diagnosis of colonic perforation and obstruction. The decision was made to transfer the patient to a hospital near her home for palliative care and to discontinue aggressive treatment. However, her condition deteriorated, and she died within 1 day of the transfer. After the patient died, *A. finegoldii* was detected in the blood culture obtained during an episode of septic shock.

The patient in fatal case 8 was a man in his 70s who was hospitalized for a comprehensive evaluation of adenocarcinoma of unknown primary origin. The patient received fluconazole for oral candidiasis. During hospitalization, he experienced septic shock and received intravenous fluids. Blood cultures were obtained, and TZP was administered. Although the patient initially stabilized, he later experienced fecal emesis, likely caused by intestinal obstruction. One day after onset, he died of septic shock. Blood cultures obtained during a septic shock episode revealed the presence of *A. onderdonkii*.

### Microbiologic Characteristics

**Figure 1:**
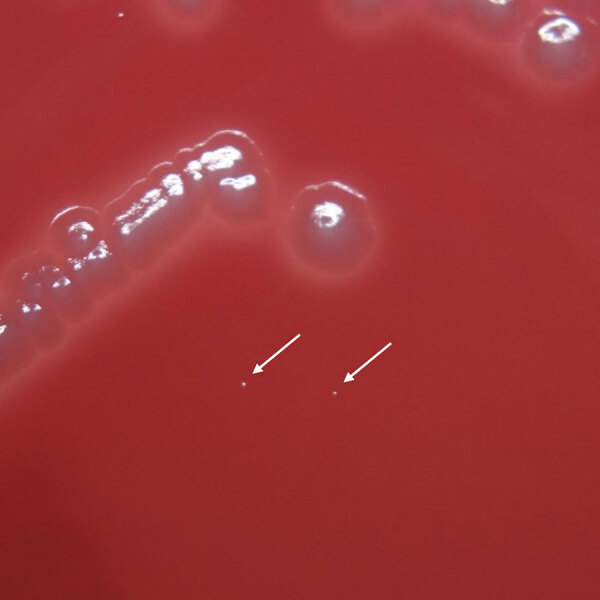
Small colonies of *Alistipes finegoldii* grown on agar medium in anaerobic culture in study of *Alistipes* bacteremia in a tertiary care center, Japan, 2016–2023. *A. finegoldii* (arrows) and *Escherichia coli* ATCC 25922 colonies were grown on Brucella HK agar medium after 2 days of anaerobic culture. The colonies of *A. finegoldii* were notably smaller in size than those of *E. coli* ATCC 25922.

**Figure 2:**
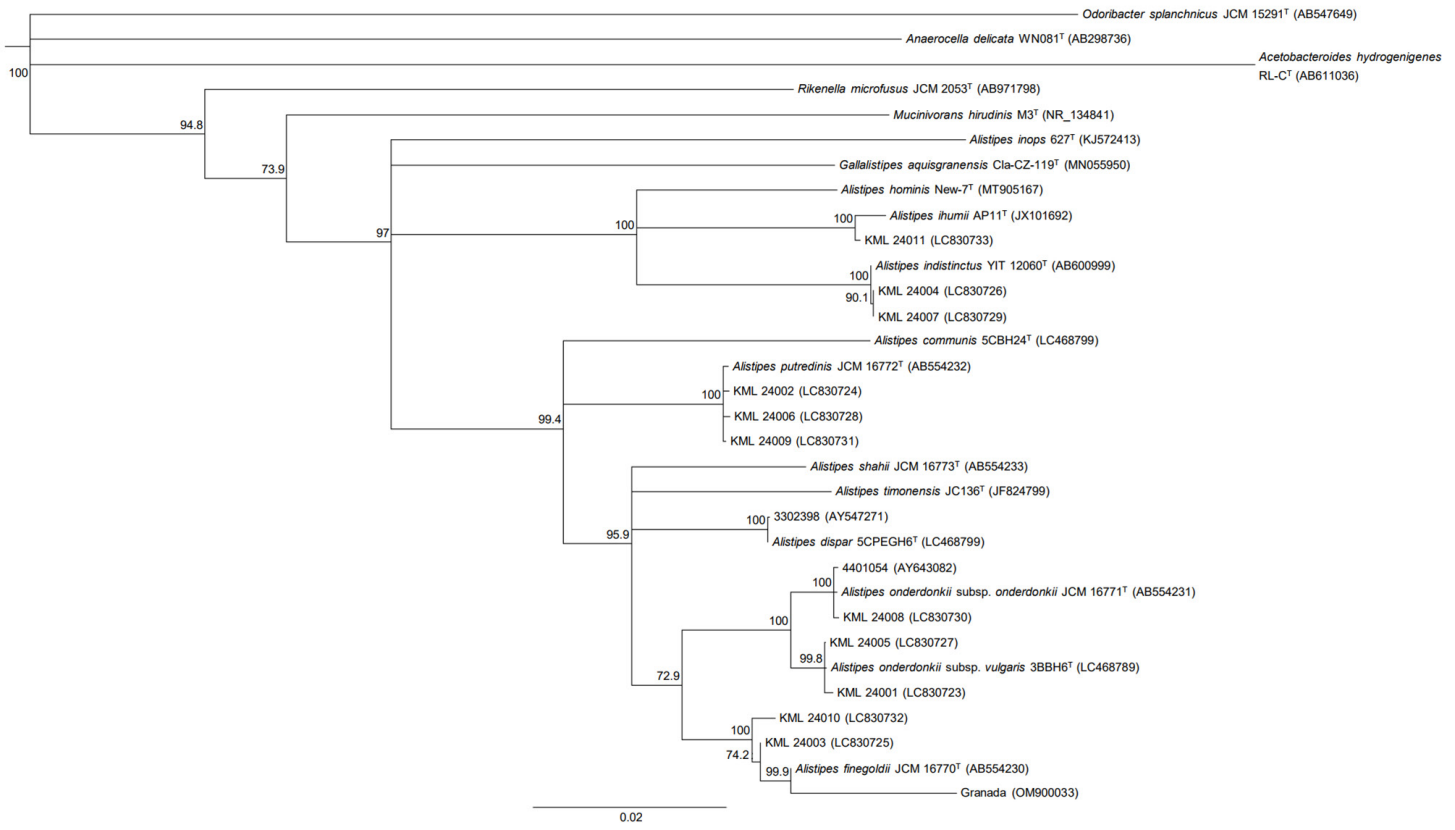
Phylogenetic tree of strains in the genus *Alistipes* based on 16S rRNA gene sequences in study of *Alistipes* bacteremia in a tertiary care center, Japan, 2016–2023. Tree illustrates evolutionary relationships between strains in the family *Rikenellaceae* and the genus *Alistipes*. GenBank accession numbers appear in parentheses. Numbers at branch nodes are bootstrap values (>70%) based on 1,000 replicates for both the neighbor-joining and maximum likelihood methods. Scale bar represents 0.02 substitutions per nucleotide position.

*Alistipes* strains formed small colonies on the Brucella HK agar medium (Figure 1). MALDI-TOF mass spectrometry identified 13 strains with species-level accuracy, including *A. finegoldii* (n = 4), *A. onderdonkii* (n = 4), *A. putredinis* (n = 3), and *A. indistinctus* (n = 2) (Table 3; Figure 2). Of the 13 strains, 10 preserved strains underwent 16S rRNA gene analysis and WGS. We excluded the 3 nonpreserved strains from our analysis. 16S rRNA gene analysis showed that the 10 preserved strains were 99.8%–100% similar to the species identified by MALDI-TOF mass spectrometry (Appendix Table 2). 

**Table 3:** Identification results of analyses for *Alistipes *strains isolated from patients with *Alistipes* bacteremia, Japan, 2016–2023*

Case no.	Year	Strain name	MALDI-TOF MS			16S rRNA analysis			Whole-genome analysis	
			Proposed species	Score		Identified species	Similarity, %		Identified species	ANI, %
1	2016	NA	*A. finegoldii*	2.0		NA	NA		NA	NA
2	2017	NA	*A. onderdonkii*	2.1		NA	NA		NA	NA
3	2018	NA	*A. finegoldii*	2.1		NA	NA		NA	NA
4	2019	KML 24001	*A. onderdonkii*	2.3		*A. onderdonkii*	99.9		*A. onderdonkii*	98.3
5	2019	KML 24002	*A. putredinis*	2.1		*A. putredinis*	100		*A. putredinis*	99.9
6	2020	KML 24003	*A. finegoldii*	2.0		*A. finegoldii*	99.9		*A. finegoldii*	99.1
7	2020	KML 24004	*A. indistinctus*	2.0		*A. indistinctus*	100		*A. indistinctus*	99.1
8	2021	KML 24005	*A. onderdonkii*	2.1		*A. onderdonkii*	100		*A. onderdonkii*	99.6
9	2021	KML 24006	*A. putredinis*	2.1		*A. putredinis*	100		*A. putredinis*	99.7
10	2022	KML 24007	*A. indistinctus*	2.2		*A. indistinctus*	100		*A. indistinctus*	99.2
11	2022	KML 24008	*A. onderdonkii*	2.0		*A. onderdonkii*	100		*A. onderdonkii*	98.8
12	2023	KML 24009	*A. putredinis*	2.1		*A. putredinis*	99.9		*A. putredinis*	98.9
13	2023	KML 24010	*A. finegoldii*	2.3		*A. finegoldii*	99.8		NA	NA
13	2023	KML 24011	No identification	NA		*A. ihumii*	99.7		NA	NA

*ANI, average nucleotide identity; MALDI-TOF MS, matrix-assisted laser desorption/ionization time-of-flight mass spectrometry; NA, not available.

The draft genomes of 9 strains in our study (KML 24001–24009) had contamination rates of <1% and were used for further analysis (Appendix Table 3). One remaining strain had a contamination rate >5% and was excluded from further analysis. The genome size of *A. finegoldii *was 3.6 Mb; of *A. onderdonkii*, 3.3–3.6 Mb; of *A. putredinis*, 2.5–2.8 Mb; and *A. indistinctus*, 2.8–2.9 Mb (Appendix Table 4). The ANI results of KML 24001–24009 were consistent with the results of MALDI-TOF mass spectrometry, with an ANI of 99.8%–100% (Appendix Table 5). DDH analysis revealed that KML 24001 and KML 24005 exhibited DDH values of >80% only with *A. onderdonkii* subspecies *vulgaris* 3BBH6^T^. In contrast, KML 24008 exhibited DDH values of >80% only with *A. onderdonkii* subsp. *onderdonkii* DSM 19147^T^ (Appendix Table 6).

We purified strains with contamination rate of >5% through subculturing and reidentified the 2 types of colonies using MALDI-TOF mass spectrometry and 16S rRNA gene analysis. We identified 1 strain (KML 24010) as *A. finegoldii* using MALDI-TOF mass spectrometry; it exhibited 99.8% similarity to *A. finegoldii* DSM 17242^T^ based on 16S rRNA gene analysis. Another strain (KML 24011) could not be identified using MALDI-TOF mass spectrometry and exhibited 99.7% similarity to *A. ihumii* AP11^T^.

Reanalysis of the 3 *A. finegoldii* strains isolated in previous reports yielded the following results: strain 3302398 showed 100% similarity to *Alistipes dispar* 5CPEGH6^T^, and strain 4401054 showed 99.0%–100% similarity to *A. onderdonkii* subsp. *onderdonkii* DSM 19147^T^. Strain Granada showed 97.6% similarity with *A. finegoldii* DSM 17242^T^. The 16S rRNA gene sequences and draft genome data have been deposited in GenBank via the DNA DataBank of Japan (Appendix Tables 2, 4).

### Antimicrobial Susceptibility and Resistance Genes

**Figure 3:**
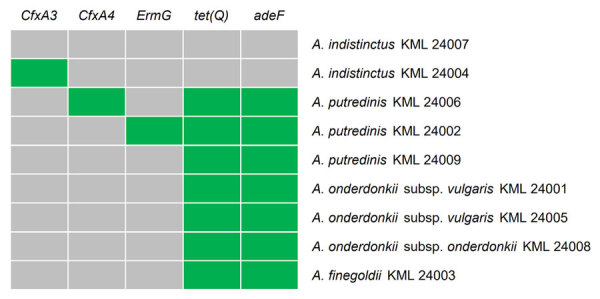
Heatmap representing distribution of antimicrobial resistance genes in *Alistipes* strains in study of *Alistipes* bacteremia in a tertiary care center, Japan, 2016–2023. Horizontal axis shows resistance genes; vertical axis shows strains. Gray cells indicate the absence of a resistance gene, and green cells indicate its presence.

Antimicrobial susceptibility data were available for 10 *Alistipes* strains (Table 4). Low MIC_90_ values were observed for SAM (2 μg/mL), AMC (2 μg/mL), TZP (<4 μg/mL), IPM (1 μg/mL), CLI (1 μg/mL), and MTZ (0.5 μg/mL). For CLI, only 1 *A. putredinis* strain showed a MIC of 2 μg/mL, and that strain possessed the *ermG* gene (Figure 3). The drugs with high MIC_90_ values were PEN (>1 μg/mL), CRO (>32 μg/mL), FOX (32 μg/mL), MINO (8 μg/mL), and MXF (>4 μg/mL). MICs were particularly high for PEN and MINO; MIC_50_ for PEN was >1 μg/mL and for MINO 4 μg/mL. Seven strains of *A. finegoldii*, *A. onderdonkii*, and *A. putredinis* with MICs ≥4 μg/mL for MINO harbored the *adeF* and *tet(Q)* genes, and 2 strains of *A. indistinctus* showed an MIC of 32 μg/mL for FOX. Three strains, all of *A. onderdonkii,* showed MICs ≥4 μg/mL for MXF.

**Table 4:** MIC values against antimicrobial drugs and antimicrobial resistance genes of *Alistipes* strains in a tertiary care center, Japan, 2016–2023*

Strain name	PEN	SAM	AMC	TZP	CRO	FOX	IPM	CLI	MINO	MXF	MTZ	Resistance genes
Af KML 24003	0.06	<0.5	<0.25	<4	<1	<1	1	1	4†	0.5	<0.5	*adeF, tet(Q)*
Af KML 24010	0.06	<0.5	<0.25	<4	4	4	0.5	1	2	0.5	<0.5	Not tested
Ao KML 24001	>1	1	0.5	<4	8	4	1	<0.12	4†	>4	<0.5	*adeF, tet(Q)*
Ao KML 24005	0.5	<0.5	<0.25	<4	2	2	2	<0.12	8†	>4	<0.5	*adeF, tet(Q)*
Ao KML 24008	>1	2	1	<4	>32	8	1	0.5	8†	>4	<0.5	*adeF, tet(Q)*
Ap KML 24002	>1	<0.5	0.5	<4	>32	<1	<0.25	2†	4†	<0.25	<0.5	*adeF, tet(Q), ermG*
Ap KML 24006	>1	1	2	<4	>32	4†	1	<0.12	8†	1	<0.5	*adeF, tet(Q), cfxA4*
Ap KML 24009	<0.03	<0.5	<0.25	<4	<1	<1	0.5	<0.12	8†	1	<0.5	*adeF, tet(Q)*
Ain KML 24004	>1	8	8	<4	>32	32†	1	<0.12	<0.25	1	<0.5	*cfxA3*
Ain KML 24007	>1	2	1	<4	32	32	1	<0.12	<0.25	1	<0.5	Not detected
Total MIC_50_	>1	<0.5	0.5	<4	8	4	1	<0.12	4	1	<0.5	Not available
Total MIC_90_	>1	2	2	<4	>32	32	1	1	8	>4	<0.5	Not available

*MIC values are μg/mL. Af, *Alistipes finegoldii*; Ain, *Alistipes indistinctus*; AMC, amoxicillin/clavulanic acid; Ao, *Alistipes onderdonkii*; Ap, *Alistipes putredinis*; CLI, clindamycin; CRO, ceftriaxone; FOX, cefoxitin; IPM, imipenem; MIC_50_, concentration required to inhibit 50% of strains; MIC_90_, concentration required to inhibit 90% of strains_;_ MINO, minocycline; MTZ, metronidazole; MXF, moxifloxacin; PEN, penicillin; SAM, ampicillin/sulbactam; TZP, piperacillin/tazobactam. †Indicates the presence of a resistance gene for the drug within that class.

## Discussion

In this study, we identified 13 cases of *Alistipes* bacteremia at a single institution in Japan. We observed 3 main characteristics in patients with *Alistipes* bacteremia. First, most patients were elderly or had comorbid conditions; 7 of 13 patients had either solid tumors or hematologic malignancies. Second, *Alistipes* cases were generally associated with gastrointestinal diseases, and all patients were treated for true bacteremia. Third, some *Alistipes* strains had high MICs against β-lactams, tetracyclines, and quinolones, indicating the presence of antimicrobial resistance genes.

Anaerobic bacteria can cause infections in almost any part of the body, including severe bloodstream infections (*24*). In our study, 13 cases of bacteremia were caused by *A. finegoldii*, *A. putredinis*, *A. onderdonkii* subsp. *onderdonkii*, *A. onderdonkii* subsp. *vulgaris*, *A. indistinctus*, and *A. ihumii*. *Alistipes* bacteremia is a rare occurrence; only 3 cases of *A. finegoldii* have been reported to date (*11*,*12*). Tyrrell et al. identified one of these strains (strain 4401054) as *A. onderdonkii* (*7*); our data support their report. In addition, we confirmed that another strain (strain 3302398) was *A. dispar*. Compared with other bacteria, such as *E. coli* and *Bacteroides* species, *Alistipes* species grow more slowly and form significantly small colonies. Moreover, the identification of *Alistipes* species on the basis of biochemical characteristics is a challenging process. Slow growth and difficulty in identification probably contribute to the underestimation of *Alistipes* bacteremia.

The advent of MALDI-TOF mass spectrometry has improved the ability to accurately identify anaerobes, including lesser-known pathogens (*24*,*25*). Our study showed that MALDI-TOF mass spectrometry could identify *Alistipes* strains with high accuracy, in agreement with 16S rRNA gene analysis and WGS. However, the spectra database lacks coverage of all *Alistipes* species, which presents a challenge for routine identification. For example, 1 strain identified as *A. ihumii* through 16S rRNA gene analysis could not be identified using MALDI-TOF mass spectrometry. The limitations of mass spectrometry databases may contribute to the underdiagnosis of *Alistipes* infections in clinical practice. The expansion of the species database for MALDI-TOF mass spectrometry would enable more precise evaluation of *Alistipes* bacteremia.

*Alistipes* bacteremia is more likely attributable to the risk factors of patients than to the intrinsic pathogenicity of *Alistipes* species. *Alistipes* species have been isolated from feces (*3*,*6*,*26*); *A. putredinis* is commonly found in gut microbiota (*27*). In previous cases of *Alistipes* bacteremia, 2 patients had colon cancer (*11*), and another had intestinal perforation and peritonitis (*12*). In our study, most patients were older and had comorbid conditions, including solid tumors and hematologic malignancies. Six patients had polymicrobial bacteremia, indicating the infection may have originated from an anatomic breach. Gastrointestinal symptoms were observed in 11 patients, suggesting that *Alistipes* may translocate from the gastrointestinal tract into the bloodstream in patients with comorbidities and gastrointestinal abnormalities. In our cohort, all patients received antimicrobial therapy for true bacteremia, and 11 recovered. Two patients who died were >70 years of age, had solid tumors, and experienced sepsis; those patients had monomicrobial *Alistipes* bacteremia and were treated with antimicrobials to which the isolates were susceptible. The fatal outcomes were likely a result of the complications of sepsis and the effects of underlying malignancies.

*Alistipes* strains showed different antimicrobial susceptibilities depending on the species and strain. All the strains showed low MIC values for BLBLIs, IPM, and MNZ. Consistent with our strains, previously reported strains were susceptible to SAM (*7*), TZP (*12*), and MNZ (*3*,*7*,*12*). In our cohort, 12 patients were treated with BLBLIs and 10 recovered. In previous cases of *Alistipes* bacteremia, 3 patients were successfully treated with combinations of AMC and ciprofloxacin, AMC and amikacin, or TZP and MTZ (*11*,*12*). BLBLIs and MNZ are commonly used for anaerobic infections (*28*) and can be an option for treatment of *Alistipes* bacteremia.

Conversely, several *Alistipes* strains were resistant to PEN, CRO, MINO, MXF, and FOX. Low susceptibility to MXF was observed only in *A. onderdonkii* and to FOX in *A. indistinctus*, which suggests potential species-specific patterns of antimicrobial resistance. Moreover, our data revealed the presence of antimicrobial resistance genes, such as *adeF*, *tet(Q)*, *cfxA3*, *cfxA4*, and *ermG*. Consistent with our findings, previous reports have indicated resistance to PEN (*10*–*12*), tetracyclines (*3*), and MXF (*10*) in *Alistipes* strains. Although breakpoints for *Alistipes* have not been established by the Clinical and Laboratory Standards Institute (*29*), it may be prudent to avoid using antimicrobial drugs that have been observed to have low susceptibility for the treatment of *Alistipes* bacteremia.

The limitations of this study are its single-center design and the limited number of cases. Because it was a single-center study, the generalizability of patient and strain characteristics is limited. The limited number of cases for each strain prevented statistical comparisons of patient backgrounds and antimicrobial susceptibilities between the species. Therefore, future studies should include a wider range of regions and institutions. Furthermore, 2 *A. finegoldii* strains and 1 *A. onderdonkii* strain were not preserved, which restricted the validation of their identification. For the preserved strains of *A. finegoldii* and *A. onderdonkii*, the results of MALDI-TOF mass spectrometry were consistent with those of 16S rRNA gene analysis at the species level. The results suggest that the nonpreserved strains were correctly identified at the species level. We observed discrepancies between phenotypic and genotypic antimicrobial susceptibilities, and not all resistance mechanisms could be elucidated. 

In conclusion, 13 cases of *Alistipes* bacteremia were identified over a 7-year period at a tertiary care center in Japan. Most patients were elderly and had comorbid conditions involving solid tumors or hematologic malignancies. The most common symptoms were gastrointestinal, and 2 of the patients died of sepsis. Our study highlights the need for further evaluation, including mutational analysis and efflux pump assessment, to fully understand the resistance mechanisms of *Alistipes* bacteremia. Our findings suggest that *Alistipes* species can translocate from the gastrointestinal tract into the bloodstream, particularly in cases of inflammation, obstruction, or perforation, leading to severe infections. 

Appendix. Additional information about *Alistipes* bacteremia in a tertiary care center, Japan, 2016–2023.
